# *MYLIP* p.N342S polymorphism is not associated with lipid profile in the Brazilian population

**DOI:** 10.1186/1476-511X-11-83

**Published:** 2012-06-28

**Authors:** Paulo C J L Santos, Theo G M Oliveira, Pedro A Lemos, José G Mill, José E Krieger, Alexandre C Pereira

**Affiliations:** 1 Laboratory of Genetics and Molecular Cardiology, Heart Institute (InCor), University of Sao Paulo Medical School, Sao Paulo, Brazil; 2 Hemodynamic Laboratory, Heart Institute (InCor), University of Sao Paulo Medical School, Sao Paulo, Brazil; 3 Department of Physiology, Espirito Santo Federal University, Espirito Santo, Brazil

**Keywords:** *MYLIP*, p.N342S, rs9370867, Lipid profile, Cholesterol, Ethnicity, Brazilian

## Abstract

**Background:**

A recent study investigated the *MYLIP* region in the Mexican population in order to fine-map the actual susceptibility variants of this *locus*. The p.N342S polymorphism was identified as the underlying functional variant accounting for one of the previous signals of genome-wide association studies and the N342 allele was associated with higher cholesterol concentrations in Mexican dyslipidemic individuals. To date, there is no further evaluation on this genotype-phenotype association in the literature. In this scenario, and because of a possible pharmacotherapeutic target of dyslipidemia, the main aim of this study was to assess the influence of the *MYLIP* p.N342S polymorphism on lipid profile in Brazilian individuals.

**Methods:**

1295 subjects of the general population and 1425 consecutive patients submitted to coronary angiography were selected. General characteristics, biochemical tests, blood pressures, pulse wave velocity, and coronary artery disease scores were analyzed. Genotypes for the *MYLIP* rs9370867 (p.N342S, c.G1025A) polymorphism were detected by high resolution melting analysis.

**Results:**

No association of the *MYLIP* rs9370867 genotypes with lipid profile, hemodynamic data, and coronary angiographic data was found. Analysis stratified by hyperlipidemia, gender, and ethnicity was also performed and the sub-groups presented similar results. In both general population and patient samples, the *MYLIP* rs9370867 polymorphism was differently distributed according to ethnicity. In the general population, subjects carrying GG genotypes had higher systolic blood pressure (BP), diastolic BP, and mean BP values (129.0 ± 23.3; 84.9 ± 14.6; 99.5 ± 16.8 mmHg) compared with subjects carrying AA genotypes (123.7 ± 19.5; 81.6 ± 11.8; 95.6 ± 13.6 mmHg) (p = 0.01; p = 0.02; p = 0.01, respectively), even after adjustment for covariates. However, in analysis stratified by ethnicity, this finding was not found and there is no evidence that the polymorphism influences BP.

**Conclusion:**

Our findings indicate that association studies involving this *MYLIP* variant can present distinct results according to the studied population. In this moment, further studies are needed to reaffirm if the *MYLIP* p.N342S polymorphism is functional or not, and to identify other functional markers within this gene.

## Background

Lipid profile disorders have been significantly associated with risk of cardiovascular disease (CVD), which is also influenced by genetic factors, hypertension, type 2 diabetes mellitus, obesity, and smoking. CVD are the main cause of morbidity and mortality in developed countries and the financial cost is enormous. Thus, guidelines from the National Cholesterol Education Program (NCEP) rely on low-density lipoprotein cholesterol (LDL-C) for the prevention of CVD [1-6].

A conventional lipid panel reports several parameters, including total cholesterol (TC), LDL-C, high-density lipoprotein cholesterol (HDL-C), and triglycerides. Of these, the NCEP and the American Heart Association recommend using LDL-C as a primary target of therapy in conjunction with assessing cardiovascular risk factors. The Third Report of the Expert Panel on Detection, Evaluation, and Treatment of High Blood Cholesterol in Adults (Adult Treatment Panel III, or ATP III - NCEP) updated clinical guidelines for cholesterol testing such as high TC (≥ 240 mg/dL), high LDL-C (≥ 160 mg/dL), and low HDL-C (< 40 mg/dL) [3,7,8].

Population genetic and epidemiological studies could help to assess the etiologic role of lipid profile in CVD and, novel genetic determinants of blood lipids can also help to provide new insights into the biological pathways and identify novel therapeutic targets. In this way, current genome-wide association studies (GWAS) have identified genetic loci contributing to inter-individual variation in serum concentration of lipids [9-12]. Some GWAS, using cohorts of mixed European descent, identified non-coding polymorphisms in the region of the *MYLIP* gene that were associated with LDL-C concentrations [12-14]. The *MYLIP* functional variant and the mechanistic basis of these associations were recently postulated by Weissglas-Volkov et al. [15].

*MYLIP* gene encodes a regulator of the LDL receptor pathway for cellular cholesterol uptake called MYLIP (myosin regulatory light chain interacting protein; also known as IDOL). Weissglas-Volkov et al. investigated the *MYLIP* region in the Mexican population in order to fine-map the actual susceptibility variants. They identified the rs9370867 non-synonymous polymorphism (p.N342S) as the underlying functional variant accounting for one of the previous GWAS significant signals and associated N342 allele with higher TC concentrations in Mexican dyslipidemic individuals [15].

To date, there is no further evaluation on this genotype-phenotype association (*MYLIP* p.N342S – lipid profile). In this scenario, the main aim of this study was to assess the influence of the *MYLIP* polymorphism on lipid profile in Brazilian individuals.

## Methods

### General population

One thousand two hundred ninety-five subjects of the general urban population were selected from Vitoria, Brazil [16]. The study design was based on cross-sectional research methodology and was developed by means of surveying and analyzing socioeconomic and health data in a probabilistic sample of residents from the municipality of Vitoria, Espirito Santo, Brazil. The sampling plan had the objective of ensuring that the research would be socioeconomically, geographically, and demographically representative of the residents of this municipality. The study protocol was approved by the involved Institutional Ethics Committees and written informed consent was obtained from all participants prior to enter the study.

### Patients submitted to coronary angiography

One thousand four hundred twenty-five consecutive patients submitted to coronary angiography for the first time to study suggestive coronary artery disease etiology were selected at the Laboratory of Hemodinamics, Heart Institute (Incor), Sao Paulo, Brazil. All patients had a clinical diagnosis of angina pectoris and stable angina. No patient enrolled in this study was currently experiencing an acute coronary syndrome. Patients with previous acute ischemic events, heart failure classes III–IV, hepatic dysfunction, familiar hypercholesterolemia, previous heart or kidney transplantation, and in antiviral treatment were excluded [17-19]. Patients answered a clinical questionnaire that covered questions regarding personal medical history, family antecedents of CVD, sedentarism, smoking status, hypertension, obesity, dyslipidemia, diabetes, and current treatment. All patients signed an informed consent form and the study has been approved by the local Ethics committee.

### Demographic data and laboratory tests

Weight and height were measured according to a standard protocol, and body mass index (BMI) was calculated. Individuals answered a clinical questionnaire that covered questions regarding smoking status and current medical treatment. Individuals who had ever smoked more than five cigarettes per day for the last year were classified as smokers [1,20]. Ethnicity was classified with a validated questionnaire for the Brazilian population according to a set of phenotypic characteristics (such as skin color, hair texture, shape of the nose and aspect of the lip) and individuals were classified as White, Intermediate (meaning Brown, *Pardo* in Portuguese), Black, Amerindian or Oriental descent [16,21,22].

Triglycerides (TG), TC, HDL-C, LDL-C, and glucose were evaluated by standard techniques in 12-h fasting blood samples. Diabetes mellitus was diagnosed by the presence of fasting glucose ≥ 126 mg/dL or the use of antidiabetic drugs [23]. Hyperlipidemia was defined as TC ≥ 240 mg/dL, LDL-C ≥ 160 mg/dL, and/or use of hypolipidemic drugs [7].

### Blood pressure phenotypes

Blood pressure was measured in the sitting position with the use of a standard mercury sphygmomanometer on the left arm after 5 min rest. The first and fifth phases of Korotkoff sounds were used for systolic blood pressure (SBP) and diastolic blood pressure (DBP), respectively. The SBP and DBP were calculated from two readings with a minimal interval of 10 min apart. Hypertension was defined as mean SBP ≥140 mmHg and/or DBP ≥90 mm Hg and/or antihypertensive drug use [24]. The mean blood pressure (MBP) was calculated as the mean pulse pressure added to one-third of the DBP.

### Pulse wave velocity and arterial stiffness

Carotid-femoral pulse wave velocity (PWV) was analyzed with an automatic device (Complior®; Colson) by an experienced observer blinded to clinical characteristics. Briefly, common carotid artery and femoral artery pressure waveforms were recorded non-invasively using a pressure-sensitive transducer (TY-306-Fukuda®; Fukuda; Tokyo, Japan). The distance between the recording sites (D) was measured, and PWV was automatically calculated as PWV = D/t, where (t) means pulse transit time. Measurements were repeated over 10 different cardiac cycles, and the mean was used for the final analysis. The validation and its reproducibility have been previously described, and increased arterial stiffness was defined as PWV ≥ 12 m/s [16,25].

### Coronary artery disease scores

Twenty coronary segments were scored: each vessel was divided into three segments (proximal, medial, and distal), except for the secondary branches of the right coronary artery (posterior ventricular and posterior descending), which were divided into proximal and distal segments. Stenosis higher than 50% in any coronary segment was graded 1 point and the sum of points for all 20 segments constituted the Extension Score. Lesion severity was calculated as follows: none and irregularities, 0 points; <50%, 0.3 points; 50–70%, 0.6 points; >70–90%, 0.8 points; and >90–100%, 0.95 points. The Severity Score was calculated through the sum of points for all 20 coronary segments [17].

### Genotyping

Genomic DNA from subjects was extracted from peripheral blood following standard salting-out procedure. Genotypes for the *MYLIP* rs9370867 (p.N342S, c.G1025A) polymorphism was detected by polymerase chain reaction (PCR) followed by high resolution melting (HRM) analysis with the Rotor Gene 6000® instrument (Qiagen, Courtaboeuf, France) [26,27]. The QIAgility® (Qiagen, Courtaboeuf, France), an automated instrument, was used according to instructions to optimize the sample preparation step. One specific disc is able to genotype 96 samples for this polymorphism [28].

Amplification of the fragment was performed using the primer sense 5’- TTGTGGACCTCGTTTCAAGA -3’ and antisense 5’- GCTGCAGTTCATGCTGCT -3’ (80 pairs base) for the rs9370867. A 40-cycle PCR was carried out with the following conditions: denaturation of the template DNA for first cycle of 94°C for 120 s, denaturation of 94°C for 20 s, annealing of 53.4°C for 20 s, and extension of 72°C for 22 s. PCR was performed using a 10 μL reactive solution (10 mM Tris–HCl, 50 mM KCl, pH 9.0; 2.0 mM MgCl_2_; 200 μM of each dNTP; 0.5 U Taq DNA Polymerase; 200 nM of each primer; 10 ng of genomic DNA template) with addition of fluorescent DNA-intercalating SYTO9® (1.5 μM; Invitrogen, Carlsbad, USA).

**Figure 1:**
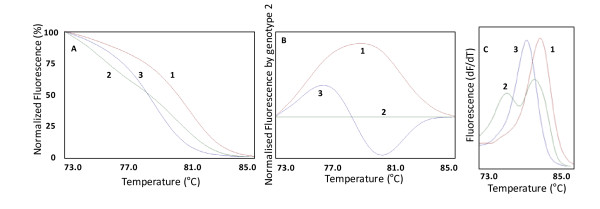
**Graphs of the *MYLIP *rs9370867 (p.N342S, c.G1025A) nucleotide changes results in different curve patterns using high resolution melting analysis. A**: Graph of normalized fluorescence by temperature. **B**: Graph of normalized fluorescence (based on genotype 2) by temperature. **C**: Graph of melting curve analysis (fluorescence differential/temperature differential). 1: wild-type genotype (GG); 2: heterozygous genotype (GA); 3: mutant homozygous genotype (AA).

In the HRM phase, the Rotor Gene 6000® measured the fluorescence in each 0.1°C temperature increase in the range of 73-85°C. Melting curves were generated by the decrease in fluorescence with the increase in the temperature; and in analysis, nucleotide changes result in three different curve patterns (Figure 1). Samples of the three observed curves were analyzed using bidirectional sequencing as a validation procedure (ABI Terminator Sequencing Kit® and ABI 3500XL Sequencer® - Applied Biosystems, Foster City, CA, USA). The two methods gave identical results in all tests. The wild-type, heterozygous and mutant homozygous genotypes for the rs9370867 could be easily discernible by HRM analysis. In addition, 4% of the samples were randomly selected and reanalyzed as quality controls and gave identical results.

### Statistical analysis

Categorical variables are presented as percentage while continuous variables are presented as mean ± standard deviation. Chi-square test was performed for comparative analysis of gender, ethnicity, hypertension, diabetes, hyperlipidemia, increased arterial stiffness, and smoking frequencies according to *MYLIP* polymorphism. Chi-square test was also performed for the Hardy-Weinberg equilibrium. ANOVA test was performed for comparing the age, BMI, biochemical data, blood pressures, PWV, and angiographic data means according to *MYLIP* polymorphism. Tukey's *post hoc* test was performed to identify the different group. Biochemical data, blood pressures, and angiographic data were adjusted for age, gender, and ethnicity. PWV was adjusted for age, gender, MBP, and ethnicity.

The analysis stratified by hyperlipidemia was performed according to Weissglas-Volkov et al.‘s inclusion and exclusion criteria [8,15]. Fasting serum TG > 200 mg/dL for the cases, TG < 150 mg/dL for the controls, and subjects with morbid obesity (BMI > 40 kg/m^2^) or type 2 diabetes mellitus were excluded. The subjects were classified as high-TC if their serum TC levels were ≥ 240 mg/dL and normal TC if their serum TC levels were < 240 mg/dL in the absence of lipid lowering medication. The subjects were also classified as combined hyperlipidemia if their serum TC and TG levels were ≥ 240 mg/dL and > 200 mg/dL, and as controls if TC and TG levels were < 240 mg/dL and < 150 mg/dL, respectively [8,15]. All statistical analyses were carried out using the SPSS software (v. 16.0), with the level of significance set at p < 0.05.

## Results

### General characteristics according to MYLIP polymorphism

Table 1 summarizes general characteristics of both studied samples.

**Table 1:** **General characteristics according to*MYLIP*rs9370867 genotypes**

**General population**	**Genotypes**			***p*value**
**(n= 1295, 100%)**	**GG (n= 547)**	**GA (n= 571)**	**AA (n= 177)**	
Age (years)	44.1 ± 10.9	45.1 ± 10.7	45.8 ± 10.5	0.14
**Gender, female** (%)	52.7	54.1	55.9	0.72
**Ethnicity**^**a**^ (%)				
White	30.7	47.2	22.1	
Intermediate	45.2	44.4	10.4	<0.001
Black	72.7	24.2	3.1	
Other	47.9	45.3	6.8	
**Hypertension**^**b**^ (%)	48.6	45.2	36.1	0.05
**Diabetes**^**c**^ (%)	6.4	8.1	6.2	0.49
**Hyperlipidemia**^**d**^ (%)	26.3	27.1	27.7	0.33
**Increased arterial stiffness**^**e**^ (%)	16.1	14.9	10.2	0.20
**Smokers**^**f**^ (%)	24.9	24.5	24.3	0.58
**Body mass index**^**g**^ (Kg/m^2^)	26.4 ± 5.2	26.3 ± 4.7	25.9 ± 4.5	0.60
Patients submitted to coronary angiography	Genotypes			*p* value
(n= 1425, 100%)	GG (n= 545)	GA (n= 633)	AA (n= 247)	
**Age** (years)	59.5 ± 10.6	60.2 ± 10.1	60.1 ± 10.3	0.55
**Gender, female** (%)	41.7	41.2	36.0	0.29
**Ethnicity** (%)				
White	31.6	47.7	20.7	
Intermediate	44.8	42.3	12.9	<0.001
Black	63.2	30.9	5.9	
Other	91.2	8.8	0	
**Hypertension** (%)	74.6	73.4	67.1	0.08
**Diabetes** (%)	35.4	34.1	34.2	0.56
**Hyperlipidemia** (%)	57.6	61.1	56.2	0.69
**Smoker** (%)	32.7	37.0	38.1	0.21
**Body mass index** (Kg/m^2^)	27.8 ± 4.9	27.6 ± 4.7	27.7 ± 4.9	0.67

^a^Ethnicity was categorized in White, Black, Intermediate (person with admixture between White and Black) and other (Amerindians and Oriental descent).

^b^Hypertension was defined as mean systolic blood pressure ≥ 140 mmHg and/or diastolic blood pressure ≥ 90 mmHg and/or use of anti-hypertension drugs.

^c^Diabetes was defined as fasting glucose ≥ 126 mg/dL and/or use of hypoglycemic drugs.

^d^Hyperlipidemia was defined as total-cholesterol ≥ 240 mg/dL, low density lipoprotein-cholesterol ≥ 160 mg/dL, and/or use of hypolipidemic drugs.

^e^Increased arterial stiffness was defined as pulse wave velocity ≥ 12 m/s.

^f^Individuals who had ever smoked more than five cigarettes per day for the last a year were classified as smokers.

^g^Adjusted for age and gender.

No difference in the frequencies of hyperlipidemia, diabetes, increased arterial stiffness, and smoking status according to *MYLIP* polymorphism was found. Only hypertension frequency presented a trend (p = 0.05) in the general population (Table 1).

In both general population and patient samples, the *MYLIP* rs9370867 polymorphism was differently distributed according to ethnicity (Table 1). In the general population, the frequencies of the *MYLIP* rs9370867 A variant allele and of the homozygous genotype (AA) was higher in Whites (45.8% and 22.1%) compared with Blacks (15.2% and 3.1%) (p < 0.001 and p < 0.001, respectively). In the patients submitted to coronary angiography, the frequencies of the *MYLIP* rs9370867 A variant allele and of the homozygous genotype (AA) was higher in Whites (44.6% and 20.7%) compared with Blacks (21.3% and 5.9%) (p < 0.001 and p < 0.001, respectively). The genotypic distribution for the *MYLIP* rs9370867 polymorphism was in Hardy–Weinberg equilibrium according to ethnic groups.

### Biochemical, hemodynamic, and angiographic data according to MYLIP polymorphism

Table 2 summarizes biochemical, hemodynamic, and angiographic data of both studied samples.

**Table 2:** **Biochemical, hemodynamic, and angiographic data according to*MYLIP*rs9370867 genotypes**

**General population**	**Genotypes**			***p*value**
**(n= 1295, 100%)**	**GG (n= 547)**	**GA (n= 571)**	**AA (n= 177)**	
**Total cholesterol** (mg/dL)	213 ± 42.9	215.7 ± 45.8	213.5 ± 41.1	0.56
**LDL-C** (mg/dL)	142.3 ± 37.4	143.6 ± 40.8	140.9 ± 36.5	0.67
**HDL-C** (mg/dL)	46.0 ± 13.0	45.5 ± 12.1	45.9 ± 12.7	0.77
**Triglycerides** (mg/dL)	126.9 ± 87.1	141.3 ± 138.9	135.6 ± 95.2	0.10
**Glycemia** (mg/dL)	105.0 ± 30.2	104.3 ± 30.0	101.2 ± 27.1	0.33
**Systolic blood pressure** (mmHg)	129.0 ± 23.3^a^	127.2 ± 21.3^a,b^	123.7 ± 19.5^b^	0.01
**Diastolic blood pressure** (mmHg)	84.9 ± 14.6^a^	83.9 ± 13.9^a,b^	81.6 ± 11.8^b^	0.02
**Mean blood pressure** (mmHg)	99.5 ± 16.8^a^	98.3 ± 15.5^a,b^	95.6 ± 13.6^b^	0.01
**Pulse wave velocity** (m/s)	9.9 ± 2.3	9.9 ± 2.2	9.7 ± 1.9	0.15
Patients submitted to coronary angiography	Genotypes			*p* value
(n= 1425, 100%)	GG (n= 545)	GA (n= 633)	AA (n= 247)	
**Total cholesterol** (mg/dL)	228.4 ± 48.7	232.5 ± 51.4	228.6 ± 50.2	0.39
LDL-C (mg/dL)	148.1 ± 45.0	149.6 ± 41.3	147.5 ± 48.8	0.86
HDL-C (mg/dL)	43.0 ± 12.4	42.4 ± 11.5	42.0 ± 11.4	0.64
Triglycerides (mg/dL)	184.0 ± 124.6	182.3 ± 147.6	188.1 ± 147.7	0.87
Glycemia (mg/dL)	128.0 ± 50.8	128.9 ± 61.0	130.9 ± 59.7	0.82
Systolic blood pressure (mmHg)	152.7 ± 31.6	150.4 ± 35.8	143 ± 38.1	0.21
Diastolic blood pressure (mmHg)	82.4 ± 17.0	82.4 ± 14.6	80.7 ± 13.1	0.78
Ejection fraction (%)	59.1 ± 15.6	61.2 ± 14.6	57.4 ± 14.9	0.17
Extension score	2.2 ± 1.7	2.2 ± 1.6	2.2 ± 1.5	0.89
Severity score	1.6 ± 1.3	1.6 ± 1.2	1.7 ± 1.3	0.97

Continuous data are expressed as mean ± standard deviation.

Values with different superscript letters are significantly different (Tukey’s *post hoc* test).

HDL-C: high density lipoprotein; LDL-C: low density lipoprotein.

Biochemical data, blood pressures, ejection fraction, and angiographic scores were adjusted for age, gender, and ethnicity.

Pulse wave velocity data was adjusted for age, gender, mean blood pressure, and ethnicity.

There was no association of the *MYLIP* rs9370867 genotypes with TC, LDL-C, HDL-C, triglycerides, and glycemia values in both samples (Table 2).

In the general population, subjects carrying GG genotypes had higher SBP, DBP, and MBP values (129.0 ± 23.3; 84.9 ± 14.6; 99.5 ± 16.8 mmHg) compared with subjects carrying AA genotypes (123.7 ± 19.5; 81.6 ± 11.8; 95.6 ± 13.6 mmHg) (p = 0.01; p = 0.02; p = 0.01, respectively), even after adjustment for age, gender, and ethnicity (Table 2). This difference was not found in the patient sample.

No association of the studied polymorphism with PWV values (available for the general population sample) or with angiographic data (extension and severity scores, available for the patient sample) was observed (Table 2).

### Analysis stratified by hyperlipidemia, gender, and ethnicity

The analysis stratified by hyperlipidemia was performed according to Weissglas-Volkov et al.‘s inclusion and exclusion criteria (see Methods section for details) [8,15]. In this analysis, the sub-groups (controls and dyslipidemic subjects) presented similar result as the total sample in both general population and patient samples, even after adjustment for covariates. In the sub-groups, no association of the variables according to genotypes was found and no difference in the variant allele frequency between sub-groups was observed (p > 0.05). In addition, the analysis stratified by gender and ethnicity did not identify significant results in both studied samples.

## Discussion

A recent study reported that *MYLIP* rs9370867 polymorphism was associated with TC levels in a Mexican dyslipidemic sample and this genetic data was supported by functional data, which demonstrated that rs9370867 influences plasma cholesterol levels by modifying the degradation of the LDL-receptor [15]. In this context and in an attempt to replicate this important association, our main finding was that the polymorphism did not influence the lipid profile in both Brazilian samples studied: general population and patients submitted to coronary angiography.

Here, the frequency of the A was much higher in Whites compared with Blacks. Previous studies reported that in African and Asian groups, the frequency is relatively low at 2% - 8%, whereas in European descent, the frequency is much higher at 49% - 60% [15,29,30]. The Brazilian population is one of the most heterogeneous in the world, and it is a mixture of different ethnic groups, composed mainly of European descent, African descent, and Amerindians. Thus, adjustement for ethnicity plus other covariates were performed and an analysis stratified by ethnicity was made. Nevertheless, no significant result was found. In this point there is a limitation: genetic markers of ancestry have not been used; however, a validated questionnaire for ethnicity classification was used.

In the same way, this variation of the allele frequency among ethnicity influenced blood pressures data in general population samples. SBP, DBP, and MBP values were higher in GG genotype group (major frequency of Blacks) while lower values were observed in AA genotype group (minor frequency of Blacks). The adjustment for covariates plus ethnicity was not able to exclude the participation of the ethnicity variable in the observed results. But, in analysis stratified by ethnicity, this finding was not found in any ethnicity group. Corroborating with this observation, our group demonstrated in a recent study that SBP, DBP, and MBP values were higher in Black individuals than in the other ethnic groups in the Brazilian general population (p < 0.001) [16]. Thus there is no evidence that the *MYLIP* studied polymorphism influences blood pressures.

In this study, the replication of the previously identified association was not found in both general population and patient samples. Two GWAS of populations of European descent have identified *MYLIP* genetic loci contributing to variation in serum lipids [12,13]. Weissglas-Volkov et al. investigated the *MYLIP* region in Mexican individuals in order to restrict the associated region and identified the variant p.N342S (rs9370867). They associated this substitution with cholesterol levels in a Mexican dyslipidemic study sample [15].

It is important to report that the mentioned study have only observed the association in Mexican dyslipidemic individuals. Our Brazilian general population sample allowed a first assessment of this association in a general population and, our second sample, patients submitted to coronary angiography, allowed an analysis with major frequency of dyslipidemic individuals. In both samples, even after performing analysis according to Weissglas-Volkov et al.‘s criteria (see Methods section), no association was observed. The patterns of linkage disequilibrium vary across populations and ethnicities according to previous studies [15,29,30]; thus, it is plausible that one or more *MYLIP* functional polymorphisms could be differently distributed leading to distinct findings.

## Conclusion

Our findings indicate that association studies involving this *MYLIP* variant can present distinct results according to the studied population. In this moment, further studies are needed to reaffirm if the *MYLIP* p.N342S polymorphism is functional or not, and to identify other functional markers in this *locus*.

## Competing interests

The authors declare that they have no competing interests.

## Authors' contributions

PCJLS carried out the molecular genetic studies, statistical analysis and drafted the manuscript. TGMO carried out the molecular genetic studies. ACP participated in the design of the study, statistical analysis and manuscript preparation. ACP, PAL, JGM, JEK participated in the design of the study and were responsible for individual selection and characterization. All authors contributed critically to the manuscript, whose present version was read and approved by all.
